# The production of high efficiency Ziegler–Natta catalyst with dual active sites nature using cyclohexyl chloride as promoter with super activity and produced superior polyethylene with controllable molecular weight distribution

**DOI:** 10.1080/15685551.2017.1394782

**Published:** 2017-11-01

**Authors:** Mehrdad Seifali Abbas-Abadi

**Affiliations:** ^a^ Polymerization Engineering, Iran Polymer and Petrochemical Institute (IPPI), Tehran, Iran

**Keywords:** Ziegler–Natta, polyethylene, cyclohexyl chloride, melt flow index, particle size distribution

## Abstract

In the previous studies, the several halocarbons (HC) were tested as promoters for a Ti-based Ziegler–Natta (ZN) catalyst at different polymerization conditions. The Results showed that chloro cyclohexane has the best operation in catalyst activity, polymer particle size growth, hydrogen responsibility and wax reduction too. For the first time in this study, the effect of Al/Ti ratio on the optimum HC/Ti ratio has been considered and the results showed that the optimum HC/Ti ratio depends on the Al/Ti ratio directly. In the optimum HC/Ti ratio, the catalyst activity and hydrogen responsibility ratio of the catalyst increase up to 125 and 55% respectively. The acceptable growth of polymer powder up to 46%, lower flow rate ratio (FRR) up to 19% and decrease of wax amount up to 12%, completed the promotion results. Furthermore, in the next part of this study and as key note, a little dose of halocarbon was used in the catalyst preparation to produce the special catalysts with dual active sites. In the catalyst preparation, the concentration of each active sites depends on the halocarbon amount and it can control the molecular weight distribution of the produced polyethylene; because each active sites have different response to hydrogen. The halocarbon based catalysts showed the remarkable effect on the catalyst activity, the molecular weight and especially molecular weight distribution (MWD). The flow rate ratio and MWD could be increased up to 77 and 88% respectively as the main result of halocarbon addition during the catalyst preparation.

## 1. Introduction

As one of the well-known Ziegler–Natta catalyst systems, MgCl_2_ (Ethoxide Type)/TiCl_4_/AlEt_3_ (TEA) is commonly used in the production of polyethylene though in the industrial HDPE and/or LLDPE plants, the different parameters are effective in the catalyst system selection [1–3]. A super active catalyst with high response to hydrogen, low wax and fouling, low co-monomer consumption for reaching a specific density and also the produced polymer powder with normal size and bulk density can solve many problems in the relative petrochemical plants [4–6].

Halocarbon is one of the possible solution for the usual problems of Ziegler–Natta catalysts. The halocarbon based polymerization system can show the superior activity and response to hydrogen, lower wax amount and grown polymer powder in the appropriate dose of halocarbon [7–9]. Although the mechanism of halocarbon effect on the catalyst system isn’t known yet but some literatures discuss on the derivation change of the catalyst center and conversion of dimeric TEA to monomeric form as halocarbon performance in the catalyst system [10–12].

The mechanism of halocarbon effect on the several studies dealing with the use of titanium and vanadium derivatives in the presence of halogenated hydrocarbon have been reported in the literature. The exact role of the halocarbon in polymerizations is unclear and is the subject of debate. In some cases, the halocarbon is thought to facilitate the reduction of the +4 centers to +3 and/or +2 states and increase the rate of polymerization. There are other examples where the catalyst is thought to be oxidized from the +2 to the +3 state by the halocarbon [13,14].

The effective interaction mode between germinal chloro groups and dimeric TEA by which the contribution of germinal chloro groups to the decomposition of dimeric TEA can be recognized. In the interaction mode, germinal chloro groups act as two-point weak Lewis base sites which fit with two Al Lewis acid sites of dimeric TEA. The specific interaction mode between dimeric TEA and germinal chloro groups is expected to accelerate the decomposition of dimeric TEA into monomeric TEA. In addition, the monomeric TEA may be somewhat stabilized by the formation of weak chloro group mediated bridge bonds. The TEA monomeric form has more activity in the production of active centers and reaction with the polymerization media poisons too [11].

Although the titanium based catalysts are well known in polyolefins, but the produced polyethylenes using these catalysts have a narrow molecular weight distribution (MWD: 4–8) and generally poor process-ability. It is very important to control the MWD and short chain branching (SCB) distribution for extrusion applications [15]. It is known that the addition of a halocarbon to the catalyst system can controls the molecular weight [7,8,16]. The previous works showed that halocarbons increase the hydrogen response and act like a chain transfer agent, decrease the molecular weight and molecular weight distribution too [7–9].

The super active catalyst with acceptable hydrogen responsibility, polymer powder growth, low wax amount, the controllable FRR containing low FRR for the injection grades and high FRR for the extrusion grades and known effective parameters can be a potential catalyst for the commercial applications.

In this work, the Ziegler–Natta heterogeneous supported catalysts were synthesized from TiCl_4_ and magnesium ethylate with MgCl_2_ *in situ* generation with and without cyclohexyl chloride. Then, (i) The effect of optimum HC/Ti ratio on the halocarbon free catalyst and the produced polyethylene’s under different Al/Ti ratios have been considered. (ii) The effect of used different amounts of cyclohexyl chloride during the catalyst preparation on the catalyst and polymer properties have been investigated.

## 2. Experimental

### 2.1. Materials

Ethylene gas (purity 99.9%) and inert gas (N_2_, purity 99.99%) were supplied by Arak petrochemical company and Roham Co (Tehran, Iran) respectively. Magnesium ethoxide, TiCl_4_ and cyclohexyl chloride chemicals were supplied by Merck chemical (Darmstadt, Germany) and were used as received. n-Heptane supplied by Arak Petrochemical and was purified over calcium hydride and stored over sodium wire and 13X and 4Å activated molecular sieves. Tri ethyl aluminum (TEA) as co-catalyst was purchased from Schering Co. (Bergkman, Germany).

### 2.2. Instruments

Brunauer–Emmett–Teller (BET) theory was used to measure the catalyst surface area, pore volume, and pore size by nitrogen adsorption and desorption measurements at 77 K using a Quanta chrome Corp. Nova 2200, Version 7.11. After catalyst sample dissolving in sulfuric acid, titanium was oxidized with hydrogen peroxide and titanium determination was done by analysis under visible spectrophotometry (*λ* = 410 nm) in a spectrophotometer from Shimadzu 6800. The chlorine amount was determined by the Volhard’s method of precipitation of AgCl with AgNO_3_. The titrometry was used to Mg determination by the catalyst acidic solution with EDTA.

Melt flow index (MFI) of polyethylene was determined under ASTM D1238 test method with a mass of 5 kg (MFI_5_) and 21.6 kg (MFI_21_) at 190 °C; the results were expressed in grams per 10 min. The FRR as flow rate ratio was referred to MFI_21_/MFI_5_ ratio and hydrogen responsibility ratio was calculated by MFI_5_ (with halocarbon)/MFI_5_ (without halocarbon).

GPC method by PL Instrument, model PL-220 was used to determine the molecular weight (Mw) and molecular weight distribution (MWD) of the produced polyethylenes. The operating conditions were set according to Ref. [17].

Particle size distribution (PSD) measurement of the produced polyethylenes were done by Sieve analysis. The sieve stack consisted of 0.01, 63, 125, 250, 425, 560, and 710 μm diameters. Sieves are normally considered to measure the distribution for sieve stack. d_0.1_, d_0.5_ and d_0.9_ mean that 10, 50, and 90% of the particles have less than or equal to the corresponding indicated particle diameter (μm), respectively. APS as average particle size calculated from normal curve and PSD span from cumulative graph as function of particle size distribution determined by

(1) $$\text{PSD}\;\text{span}=\left[{\text{d}}_{90}-{\text{d}}_{10}\right]/{\text{d}}_{50}$$

The wax amount as soluble part of the polyethylene was separated by the extraction with boiling n-heptane for 2 h by Soxhlet extraction. About 0.5 g of PE sample was put into a thimble. The insoluble part left in the thimble were dried in oven overnight to obtain the wax amount, corresponding to the proportion of material extracted from the initial polyethylene.

### 2.3. Synthesis of catalyst precursor

A four-necked flask -4 L- with a dropping funnel, a stirrer, a reflux condenser, and a thermometer equipment was used as reactor to catalyst preparation. Mg (OEt)_2_ (150 g) powder was dispersed in 2 L of a diesel oil fraction in the reactor under nitrogen gas. TiCl_4_ (600 g) was added drop-wise to this dispersion in the course of 2 h at 90 °C. The produced initial catalyst was then washed with the oil until the supernatant solution no longer contained any titanium and then dried. For the halocarbon based catalysts, all of the precursor steps are like the previous, just the halocarbon in different amounts inject to the diesel oil. The defined ratio of HC/Ti is a molar ratio in relation to the initial TiCl_4_. The analysis of the prepared catalysts are given in Table 3.

### 2.4. Polymerization experiments

A 1 L stirred Buchi stainless steel reactor was used to ethylene polymerizations under slurry conditions at constant temperature (83 °C) and pressure (8.5 bar). A Huber circulator, model Polysat CC3 was used to control the Polymerization temperature too. Reactor was purged with nitrogen gas at 94 °C for about 1.5 h to ensure the absence of moisture and oxygen, before each polymerization experiment. After cooling the reactor to 83 °C, the reactor was fed with 500 mL of dry heptane and then -with stirring- a given Al/Ti ratio of TEA, cyclohexyl chloride and catalyst were added respectively by means of syringe in an atmosphere of purified nitrogen. The reactor was pressurized with 2 bar hydrogen and/or without hydrogen and then ethylene was fed to maintain a reactor pressure of 8.5 bar, the temperature was controlled at 83 °C, and stirrer speed was 500 rpm to minimize ethylene transport limitation. Residence time was kept constant at 1 h and at the end, the reactor was discharged and the polymer powder was dried in air.

## 3. Results and discussion

### 3.1. Results

In the previous works, some halocarbons were tested as promoters for a Titanium-based Ziegler–Natta catalyst in the slurry phase ethylene polymerization. We found out that the catalyst activity shows a peak value with the HC/Ti ratio for any studied halocarbons [7–9]. At optimum condition, the decomposition of each dimeric TEA needs to two halogen atoms simultaneously [11] and for this reason, the optimum HC/Ti ratio related to the single chlorine halocarbons is more than two chlorines halocarbons and much more than three or four chlorines halocarbons. Also the promotion curve definitely shows a peak value for any halocarbons and the optimum HC/Ti tends to zero using high chlorines halocarbons and titanium based Ziegler–Natta catalyst. In accordance with the discussion, the resultant optimum HC/Ti ratio were 128, 110, 20, 0.54, and 0.033 for cyclohexyl chloride, cycloheptyl chloride, butyl chloride, di chloro benzene and chloroform respectively [7–9]. Besides the number of chlorine atoms, the hydrocarbon type and its electro-positivity of halocarbon can affect on the optimum HC/Ti ratio and the catalyst productivity.

The molecular weight and molecular weight distribution of the produced polyethylene using Z–N catalysts can be controlled and regulated by many variables such as catalyst, co-catalyst, monomer, hydrogen, and temperature [18,19]. Hydrogen is the strongest and fastest chain transfer agent for controlling the MWD and can significantly affect the reaction rate of ethylene polymerization [20,21]. In this paper, the ethylene polymerization was studied using hydrogen as chain transfer agent in two conditions of H_2_/C_2_H_4_ = 2/6.5 bar and without hydrogen at optimum HC/Ti ratio under different Al/Ti ratios and halocarbon based catalysts. The results were just reported at the optimum points and other results eliminated. Also, the effect of halocarbons on the some properties of the obtained polymers including particle size distribution, bulk density, melt flow index (MFI) and wax amount were studied.

#### 3.1.1. The effect of Al/Ti ratio on the optimum HC/Ti ratio

The effect of increasing the Al/Ti ratio from 40 to 160 on the optimum HC/Ti ratio is given in Table 1. As shown in the table, the optimum HC/Ti depends strongly on the Al/Ti ratio directly. It may follows the mechanism of dimeric TEA decomposition to the monomeric form because each dimeric TEA molecule needs to two single chlorine halocarbons. The observations indicated that the optimum HC/Ti ratio is less than the related Al/Ti ratio amount. The laboratory experiments showed that some TEA consumes as poison scavenger and the real Al/Ti ratio is different from the initial Al/Ti ratio. As shown in Table 1, the optimum HC/Ti increases with Al/Ti ratio increasing.

**Table 1.** The effect of Al/Ti ratio on the optimum HC/Ti, catalyst yield and polymer molecular specification.

Al/Ti	HC/Ti	Catalyst yield		H_2_/C_2_H_4_ = 2/6.5						
		H_2_/C_2_H_4_ = 0/8.5	H_2_/C_2_H_4_ = 2/6.5	MFI_5_	MFI_21_	FRR	Increase in hydrogen responsibility ratio (%)	Mw*10^−5^ (g/mol)	MWD	Wax (%)
40	15	27.4	18.23	0.62	5.2	8.4	55	1.8	3.6	0.71
	0	12.3	9.55	0.40	4.1	10.3		2.2	4.2	0.78
80	50	31.2	27.25	0.64	5.7	8.9	52	1.6	3.7	0.75
	0	15.4	13.12	0.42	4.3	10.2		2.1	4.1	0.82
120	95	35.6	32.47	0.68	6.4	9.4	51	1.5	3.8	0.77
	0	19.9	16.29	0.45	4.7	10.4		1.9	4.4	0.85
160	115	38.4	34.72	0.72	7.6	10.6	44	1.3	4.5	0.76
	0	21.4	18.64	0.50	5.7	11.4		1.6	4.6	0.86

Note: P: 8.5 bar, T: 83 °C, t: 1 h, Stirrer rate: 500 rpm.

At optimum HC/Ti ratio, the catalyst yield showed enhancement up to 108 and 123% with and without hydrogen respectively in comparison with the halocarbon free systems. In the present investigation, all of the optimum HC/Ti molar ratios indicated the remarkable enhancement in the catalyst activity clearly. The Ti^III^ based active sites as the resultant active species using optimum HC/Ti ratio are in the majority in the modified catalyst. The catalyst containing said active sites have larger propagation rate and accordingly more productivity in comparison with the system without halocarbon [9].

**Figure 1.**
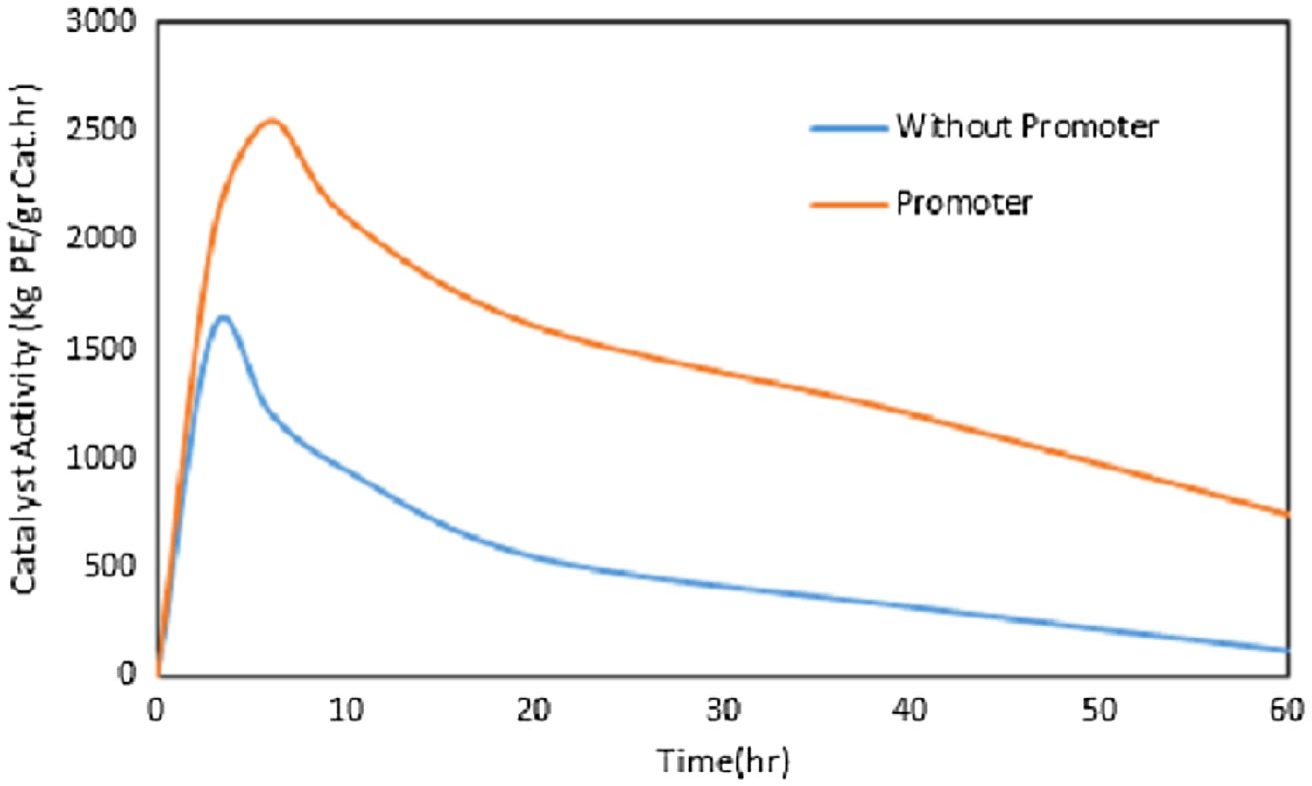
Effect of cyclohexyl chloride on the Rp (catalyst activity) during the polymerization time; HC/Ti: 0 and 95 (without organocatalyst and with organocatalyst respectively), P: 8.5 bar, T: 83 °C, Al/Ti: 120, t: 1 h, Stirrer rate: 500 rpm.

Rate of polymerization (Rp) was studied in more detail for the more efficient promoters, i.e., chloro cyclohexane. The effect of HC on the Rp during the polymerization time was illustrated in Figure 1. As can be seen, in both cases (with and without HC), after 3–4 min the catalyst activity reached a maximum peak value and then decreased with time. Therefore, it is concluded that the Rp trend has not been changed. However, in the presence of HC, rate of ethylene consumption was higher in comparison with the blank system.

The melt flow index (MFI) and flow rate ratio (FRR) as good indications of process-ability is the widely used rheological properties of polymers and occasionally the standalone rheological information used in the industry. The results of obtained polymers were characterized by MFI and GPC analysis are shown in Table 1 too. The careful observations showed that at any Al/Ti ratios, the produced polymer using optimum HC/Ti ratio has higher MFI and lower FRR, Mw and MWD values in comparison with the system without halocarbon.

As discussed, the halocarbon changes the nature of catalyst active sites especially at the optimum ratio. On the other hand, at the halocarbon free system, some catalysts sites are weak or inactive and they can’t participate in the polymerization reaction effectively. The remarkable portion of weak sites and high difference between the catalyst active sites can produce the different chains. Consequently, it is the main reason of high FRR value as symbol of broad molecular weight distribution. In the halocarbon based system, at optimum HC/Ti ratio, the Ti^III^ based active sites are in the majority and the weak sites are in the minority and as a result, the negligible portion of weak sites and lower difference between the active sites can produce the polymer with lower FRR value.

The halocarbon based system shows higher propagation and termination rates in comparison with the halocarbon free system while the increasing of termination rate is higher than the propagation rate increasing for a modified system. The catalyst system with higher propagation rate and more higher termination rate can produces the shorter chains and at close range in comparison with the system without halocarbon. The resultant polymers using optimum halocarbon showed a significant enhancement in MFI and decrease in Mw, MWD, FRR values and wax amount up to 55, 32, 17, 22 and 13% respectively. Furthermore, Al/Ti has enhancement effect on the FRR value up to 16 and 25% under the studied range of Al/Ti ratio using halocarbon and without halocarbon respectively (Table 1).

The hydrogen responsibility plays an important role in the catalyst performance. The rate of chain transfer reaction increases as function of hydrogen responsibility, resulting in the increase of MFI although hydrogen has undesirable effects such as remarkable decrease in catalyst activity and APS (Tables 1 and 2). The acceptable response to hydrogen using halocarbon up to 55% causing in the decrease of H_2_ amount to reach a certain MFI and consequently higher APS, more catalyst activity and lower catalyst consumption.

**Table 2.** The effect of Al/Ti and optimum HC/Ti ratios on the polymer powder.

Al/Ti	HC/Ti	APS (micron)	d_0.1_ (micron)	d_0.5_ (micron)	d_0.9_ (micron)	PSD span	Bulk density (g/cm^3^)
40	15	197	73.9	150.3	389.2	2.10	0.41
	0	135	64.1	111.3	215.4	1.36	0.37
80	50	206	75.6	163.7	400.4	1.98	0.41
	0	141	66.2	113.8	229.8	1.44	0.38
120	95	211	76.5	169.4	403.2	1.93	0.41
	0	154	67.4	117.6	245.1	1.51	0.39
160	115	227	78.0	187.7	428.4	1.87	0.42
	0	158	69.3	121.8	265.9	1.61	0.39

Note: P: 8.5 bar, T: 83 °C, t: 1 h, Stirrer rate: 500 rpm.

The morphology and bulk density of polymer powder plays an important role in the powder handling and separation. It is difficult to handle the puffy powder with low bulk density and/or fine powder in an industrial plant. Furthermore, the centrifuge can’t separate the powder and solvent perfectly and fines accumulate in the solvent storage tanks gradually.

**Figure 2.**
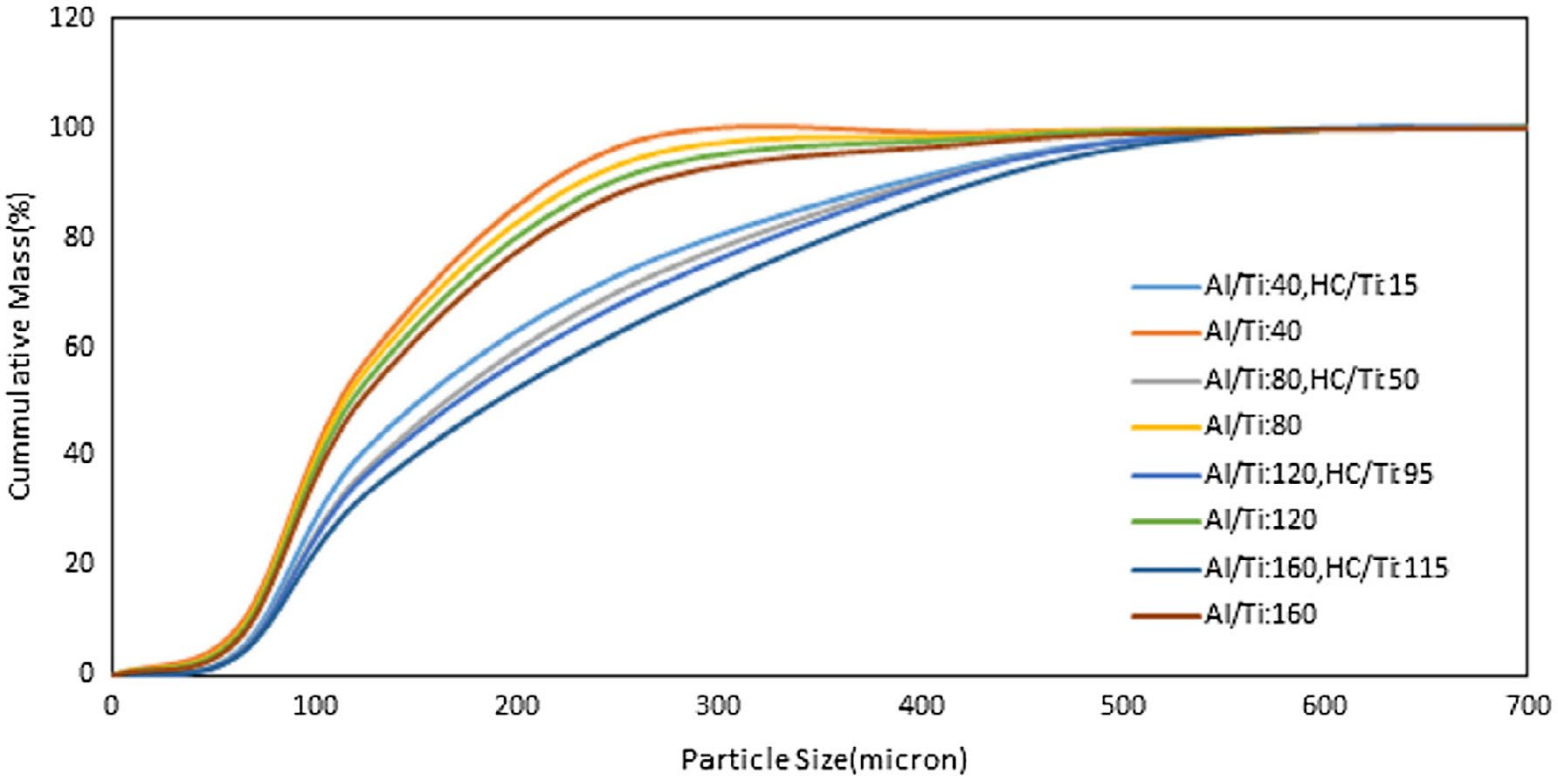
The effect of Al/Ti and optimum HC/Ti ratios on the cumulative PSD of produced PE’s P: 8.5 bar, T: 83 °C, t: 1 h, Stirrer rate: 500 rpm.

The cumulative curve of particle size distribution for catalyst system with and without halocarbon is shown in Figure 2. The cumulative distribution function of a probability distribution, evaluated at a number x, is the probability of the event that a random variable X with that distribution is less than or equal to x. d_0.1_, d_0.5_, and d_0.9_ which are good criteria to measure polymer particle size have been derived from cumulative distribution curve.

To achieve reliable and accurate PSD data for the polymer produced in all experiments, the sieve shaker method is used in this study. The results indicated that the polymer particles have remarkable growth in the presence of halocarbon due to the enhancement in the catalyst yield. According to Table 2, the significant difference between APS, d_10_, d_50_, d_90_ and bulk density of the produced polymers showed the remarkable increasing up to 46, 16, 54, 81 and 11% respectively under optimum halocarbon concentration. Also, the PSD span of the product is increased up to 55% for the said modified system.

#### 3.1.2. The effect of HC/Ti on the catalyst preparation

The effect of increasing the HC/Ti ratio from 0 to 500 ppm on the catalyst preparation is shown in Table 3. As shown, the percentage of remaining Ti in the catalytic system increased with the halocarbon increasing up to 18%. The other parameters such as APS, surface area and porosity showed little increasing in the studied range too.

**Table 3.** The effect of cyclohexyl chloride injection on the catalyst specification.

Properties value	0	50	100	200	500
Surface area (m^2^/g)	110.8	123.5	117.9	121.4	114.3
Pore volume (mL/g)	0.17	0.18	0.17	0.19	0.18
Average pore radius (Å)	17.3	15.5	16.1	16.5	15.9
APS (μm)	10.7	11.2	11.3	11.1	11.5
Ti (%)	3.8	4.2	4.3	4.1	4.5
Mg (%)	19.5	19.1	18.9	18.8	19.0
Cl (%)	56.2	56.3	55.9	56.5	55.8

The catalyst yield, Mw, MWD, melt flow index, flow rate ratio and wax content are given in Table 4. In the studied range, the observations indicated that the catalyst yield and melt flow index have uptrend and wax content has downtrend but the flow rate ratio show the maximum peak at the HC/Ti ratio of 200 ppm. At this ratio, catalyst has obvious dual active sites and the high difference between the active sites produces the wide range of polyethylene chains and increases the FRR.

**Table 4.** The effect of cyclohexyl chloride injection during the catalyst preparation on the catalyst yield and polymer molecular specification.

HC/Ti (ppm)	Catalyst yield		H_2_/C_2_H_4_ = 2/6.5					
	H_2_/C_2_H_4_ = 0/8.5	H_2_/C_2_H_4_ = 2/6.5	MFI_5_	MFI_21_	FRR	Mw*10^−5^ (g/mol)	MWD	Wax (%)
0	19.9	16.3	0.45	4.72	10.5	1.9	4.4	0.85
50	22.3	19.8	0.49	7.74	15.8	1.7	6.7	0.83
100	24.4	21.2	0.53	10.10	19.1	1.6	8.1	0.83
200	25.2	21.9	0.58	11.80	20.3	1.4	8.3	0.81
500	29.6	25.4	0.72	13.40	18.6	1.2	8.1	0.79

Note: P: 8.5 bar, T: 83 °C, Al/Ti: 120, t: 1 h, Stirrer rate: 500 rpm.

Although the catalyst activity, productivity, Mw, MWD, melt flow index and wax content as key parameters of the catalyst are important in the industrial plants but the significant difference between the FRR values and high FRR produced polyethylene by single reactor can plays determinative role in the next generations of Z–N catalysts.

The results shows that the catalyst activity increased up to 49% in the absence of hydrogen and 56% in the presence of hydrogen -2 bar- in the studied range of halocarbon. Although it seems the higher dose of halocarbon can increases the catalyst activity remarkably.

**Figure 3.**
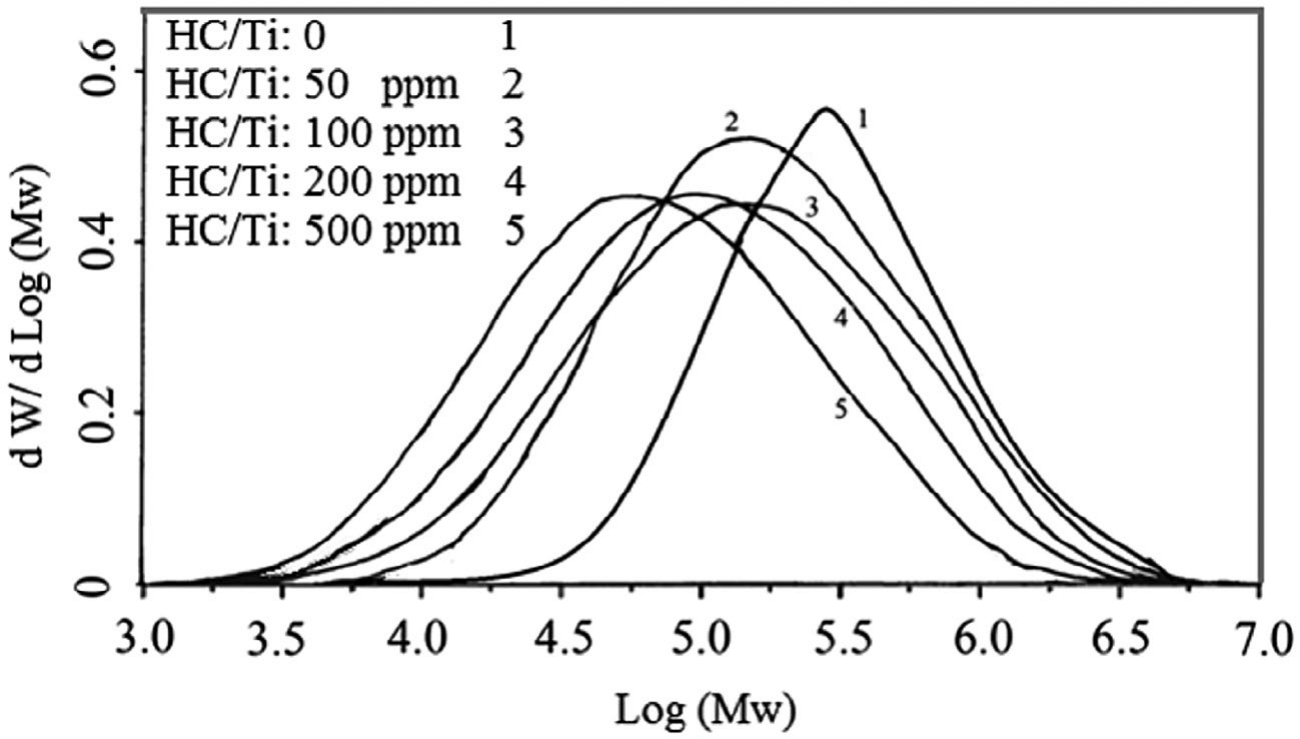
The effect of cyclohexyl chloride injection during the catalyst preparation on the molecular weight distribution of produced PE’s P: 8.5 bar, T: 83 °C, catalyst: 1 mg, Al/Ti: 120, t: 1 h, Stirrer rate: 500 rpm.

The resultant wax decreased in the used halocarbon range up to 8%. MFI and FRR as function of molecular specification showed the interesting response to the halocarbon presence in the catalyst preparation. The results indicate that MFI increased up to 60% and Mw decreased up to 37% in the studied range although higher doses of cyclohexyl chloride can affect on the Mw and MFI remarkably. In opposite with the results of halocarbon direct addition to the polymerization media, FRR and MWD values experienced a peak at the studied range and showed difference up to 77 and 84% respectively as a remarkable achievement of halocarbon based catalyst. It elucidates that the nature of active sites changes in the presence of appropriate doses of cyclohexyl chloride in the catalyst preparation. Figure 3 shows the effect of cyclohexyl chloride injection during the catalyst preparation on the molecular weight distribution of produced PE’s.

**Figure 4.**
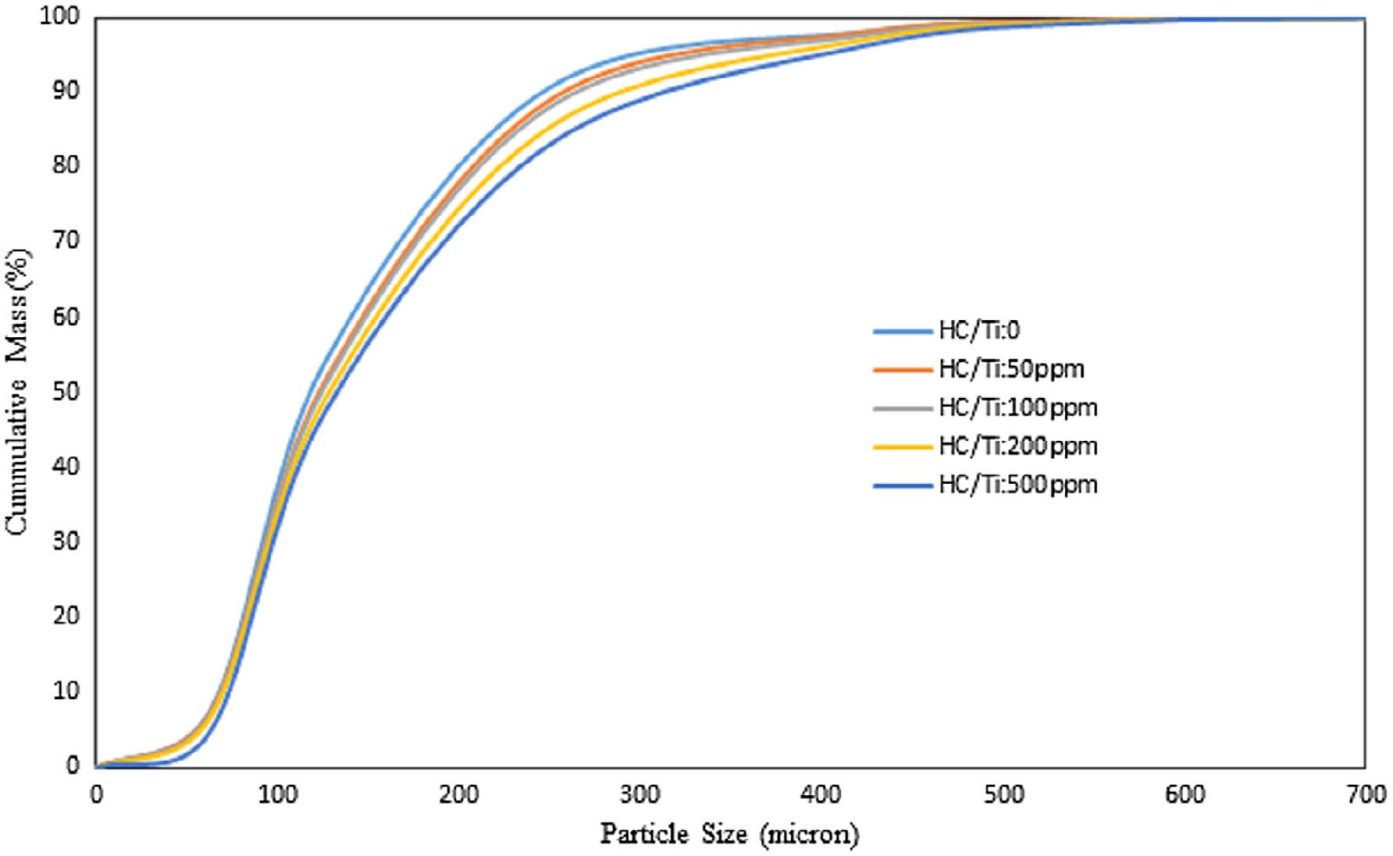
The effect of cyclohexyl chloride injection during the catalyst preparation on the cumulative PSD of produced PE’s P: 8.5 bar, T: 83 °C, catalyst: 1 mg, Al/Ti: 120, t: 1 h, Stirrer rate: 500 rpm.

Table 5 shows the particle size analysis of the produced polymers using the different doses of cyclohexyl chloride in the catalyst preparation and in the absence of hydrogen. It indicates that the polymer particle size grew in the presence of halocarbon due to the enhancement in the catalyst yield and initial catalyst APS. According to the table, APS, d_10_, d_50_, d_90_ and bulk density of the produced polymer increased up to 18, 8, 12, 27 and 8% respectively. The results showed that the halocarbon based catalyst broadened the PSD with increasing of PSD span as function of particle size distribution up to 19% in the studied range (Figure 4).

**Table 5.** The effect of cyclohexyl chloride injection during the catalyst preparation on the polymer powder.

HC/Ti (ppm)	APS	d_10_ (micron)	d_50_ (micron)	d_90_ (micron)	PSD span	Bulk density (g/cm^3^)
0	154	67.3	117.6	242.1	1.49	0.39
50	159	67.6	122.1	253.7	1.52	0.40
100	163	69.3	124.6	260.3	1.53	0.41
200	172	70.2	127.2	282.5	1.67	0.42
500	181	72.7	131.5	306.8	1.78	0.42

Note: P: 8.5 bar, T: 83 °C, Al/Ti: 120, t: 1 h, Stirrer rate: 500 rpm.

### 3.2. Discussion

The mechanism of Ziegler–Natta catalyst, halocarbon effect and other related effective parameters are not exactly known yet although the proposed mechanism can almost anticipates many phenomena. The effect of halocarbon on the active sites and the resultant products are little complex. By using a dose of halocarbon in the catalyst preparation, all of the active sites can’t react with the halocarbon molecules and as a result, some of the active sites are halocarbon based and the other active sites preserve previous theirs nature. Each active site -with different kinetic rates containing initial, propagation and termination rates- produces the special range of the polyethylene chains with different yields. Hence higher catalyst activity, lower wax amount and Mw, higher MFI, FRR and MWD values can be the conclusion of mixed active sites as dual catalyst. In other words, by using a dose of halocarbon, two different catalysts are prepared simultaneously. One of them produces the chains with lower MFI value and other catalyst produces the higher MFI polyethylene. The mix of two polymer with different MFI can produces the final polymer with high FRR value. It can be the base of new extrusion grades generation with superior process-ability and mechanical and physical properties and lower production costs.

The amount of halocarbon content can changes the share of each catalysts and the resultant polymers although the high amounts of halocarbon can produce the single halocarbon based catalyst with lower FRR and higher MFI values in comparison with the halocarbon free catalyst.

## 4. Conclusion

The effect of cyclohexyl chloride as known organocatalyst on the ethylene polymerization and catalyst preparation using Mg(OEt)_2_/TiCl_4_/TEA Ziegler–Natta catalyst system have been reported in this research. The results showed that the optimum HC/Ti ratio depend strongly on the Al/Ti ratio directly and as a result, it can be an acceptable reason for the conversion of TEA dimeric to monomeric form as halocarbon performance. In the usual range of Al/Ti ratios, the catalyst activity showed significant enhancement with and without hydrogen under optimum HC/Ti ratio. Also, the catalyst performance showed the high hydrogen responsibility, high MFI and low FRR, Mw and MWD values and wax content using the optimum concentration of halocarbon at the studied range of Al/Ti ratio.

The next section of this paper as keynote showed the effect of cyclohexyl chloride at low doses on the catalyst preparation. The results indicated that the halocarbon based catalyst acts as dual catalyst with different active site types. The new catalyst showed the enhancement in catalyst activity, MFI and APS values. The remarkable results occurred at the HC/Ti ratio of 200 ppm. The PE’s MWD using halocarbon based catalysts is 6.7–8.3 compared with the usual catalyst 4.4, broader molecular weight solved the contradiction of mechanical properties and processing performance. The resultant polymer had the high FRR and MWD values and it can open a new discussion in ethylene polymerization to produce the extrusion grades in one reactor and/or the extrusion grades with very broad molecular weight distribution and superior process-ability and mechanical properties.

## Disclosure statement

No potential conflict of interest was reported by the author.

## Funding

This work wassupported by Iran polymer and petrochemical institute.

## Supplemental data

Supplemental data for this article can be accessed https://doi.org/10.1080/15685551.2017.1394782

## Supplementary Material

TDMP_1394782_Supplementary_Material.pdf
